# High microsatellite instability (MSI-H) colorectal carcinoma: a brief review of predictive biomarkers in the era of personalized medicine

**DOI:** 10.1007/s10689-016-9884-6

**Published:** 2016-02-13

**Authors:** Zoran Gatalica, Semir Vranic, Joanne Xiu, Jeffrey Swensen, Sandeep Reddy

**Affiliations:** Caris Life Sciences, 4610 South, 44th Place, Phoenix, AZ 85040 USA; Department of Pathology, University Clinical Center of Sarajevo, Sarajevo, Bosnia and Herzegovina

**Keywords:** Colorectal cancer, Microsatellite instability, Lynch syndrome, Biomarkers, Conventional chemotherapy, Targeted therapy

## Abstract

Approximately 15 % of colorectal carcinomas (CRC) display high level microsatellite instability (MSI-H) due to either a germline mutation in one of the genes responsible for DNA mismatch repair (Lynch syndrome, 3 %) or somatic inactivation of the same pathway, most commonly through hypermethylation of the *MLH1* gene (sporadic MSI-H, 12 %). Although heterogeneous, MSI-H colorectal carcinomas as a group show some distinct biologic characteristics when compared to CRC with stable or low level microsatellite instability. In the present review we will highlight therapeutically relevant characteristics of MSI-H tumors which could lead to specific responses to some conventional chemotherapy or novel targeted therapy agents.

## Introduction

Colorectal carcinoma (CRC) represents the third most common malignancy in the developed world and one of the leading causes of cancer-related death [1]. At the molecular level, CRC is a heterogeneous disease with several molecular subtypes that harbor distinct molecular genetic, pathologic and clinical characteristics [2]. Recently, the consensus molecular subtypes (CMS) of CRC have been defined [3]. According to the new classification, four CMS with distinguishing characteristics have been proposed: CMS1 (microsatellite instability immune subtype: hypermutated subtype of CRC, microsatellite unstable with a strong immune activation); CMS2 (canonical subtype of CRC: epithelial subtype with upregulation of the WNT and MYC signaling pathways); CMS3 (metabolic subtype of CRC: epithelial subtype with metabolic dysregulation); and CMS4 [mesenchymal subtype of CRC with prominent transforming growth factor-β (TGF-β) activation, stromal invasion and neoangiogenesis] [3].

MSI refers to the hypermutable state of cells caused by impaired DNA mismatch repair (MMR). It consists of insertion and deletion mutations in stretches of short tandem DNA repeats (microsatellites) as well as nucleotide substitutions throughout the genome [4]. In this review, we simplify classification of molecular subtypes of CRC based on MSI status into two broad subgroups: MSI-high (MSI-H) and MSI-negative (low or stable) CRCs, in an effort to highlight potential therapeutic differences between these easily separable groups.

### MSI-high (MSI-H) colorectal cancer

**Fig. 1:**
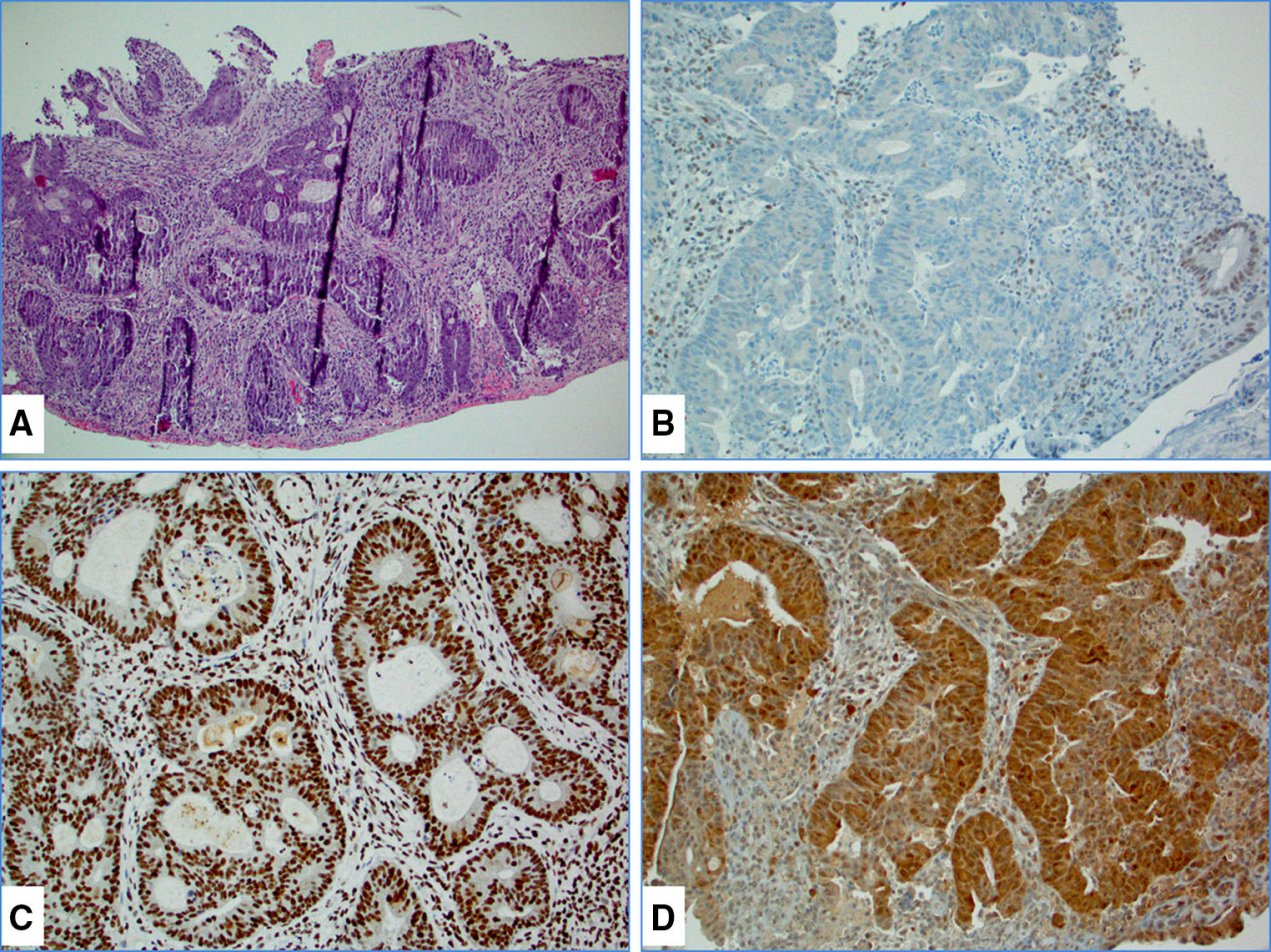
A colorectal carcinoma from a case of Lynch syndrome caused by an *MLH1* gene mutation: **A** hematoxylin and eosin (H&E) stained slide, **B** immunohistochemistry (IHC) showing concurrent loss of PMS2 in tumor cells, **C** tumor cells were diffusely positive (90–100 %) for topoisomerase 1 and **D** strongly positive (3+) for thymidylate synthase

MSI-H CRC accounts for 15 % of all CRC and includes hereditary non-polyposis colorectal cancer (HNPCC) or Lynch syndrome (3 %) and sporadic MSI-H CRC (12 %). Lynch syndrome is a highly penetrant (80 % life time risk for CRC), autosomal-dominant disorder caused by germline mutations in one of the MMR genes: *MLH1, MSH2* (70 %)*, MSH6* and *PMS2* (~30 %) [5, 6] (Fig. 1A, B). In addition, germline deletions of the last exon of the epithelial cell adhesion molecule [(*EPCAM*), a gene located upstream of MSH2] cause Lynch syndrome via epigenetic inactivation of *MSH2* [7].

**Fig. 2:**
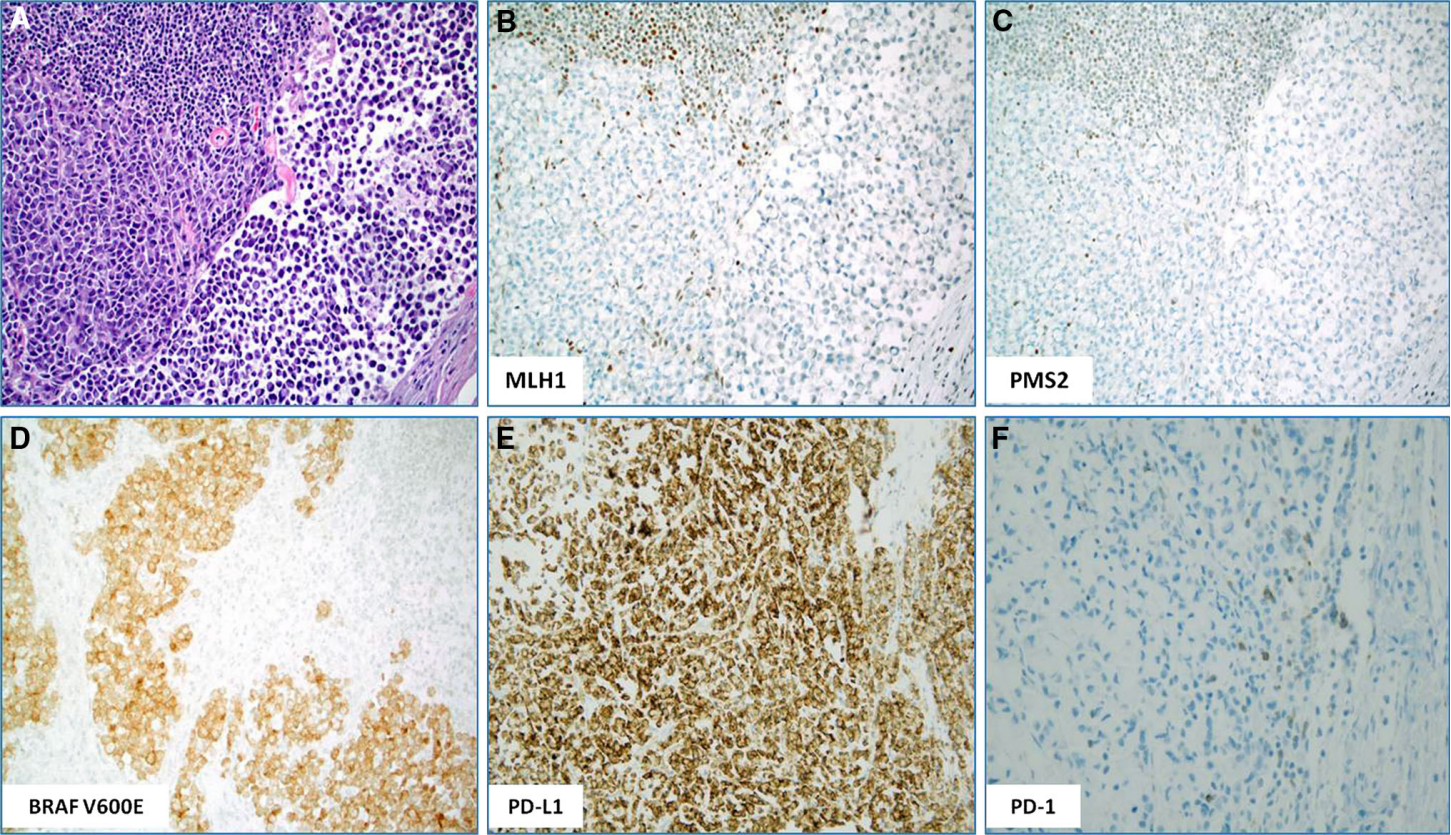
A poorly differentiated (signet ring) colorectal carcinoma with microsatellite instability-high status caused by the loss of MLH1: **A** H&E-stained slide, **B** loss of MLH1 in tumor cells by IHC, **C** concurrent loss of PMS2 in tumor cells by IHC; note retained expression of both MLH1 and PMS2 proteins in adjacent tumor-infiltrating lymphocytes, **D** IHC showing that the tumor also harbored the BRAF V600E mutation, **E** the tumor cells exhibited 2+ PD-L1 expression in ~85 % of the tumor cells (anti-PD-L1 clone SP142) and **F** while tumor infiltrating lymphocytes were positive for PD-1 protein

Sporadic MSI-H CRCs are typically caused by somatic methylation of the *MLH1* gene promoter [4] (Fig. 2A, B). It is worth noting that a small subset of MSI-H tumors harbor no alterations in the MMR genes, but overexpress various miRNAs that may silence the MMR genes. Thus, miRNA-155 downregulates MLH1, MSH2 and MSH6 mRNA, inducing MSI in CRC cell lines [8]. Similarly, miRNA-21, targeting MSH2 and MSH6 mRNA, has been found to be overexpressed in MSI-H CRC [9]. In addition, Li et al. [10, 11] found that cells lacking the SETD2 histone methyltransferase displayed microsatellite instability.

Regardless of the origin (hereditary or sporadic) or type of mutation, MSI-H CRCs share some distinct histologic cancer features (mucin-rich, signet ring and medullary types, often admixed) with increased numbers of tumor-infiltrating lymphocytes (TILs) and prominent Crohn’s-like lymphoid reaction [6, 12]. In addition, patients with Lynch syndrome have an increased risk of synchronous or metachronous tumors that include extracolonic sites (small bowel, stomach, endometrium, skin, genitourinary tract) [5, 13]. Prognostically, patients with HNPCC have a more favorable outcome (overall survival) in comparison with stage-matched sporadic CRCs [14, 15].

Methylation of the *MLH1* promoter region that is typically seen in sporadic MSI-H CRC, but not in Lynch syndrome, is strongly associated with the *BRAF* V600E gene mutation [16, 17] (Fig. 2D). In fact, presence of the *BRAF* V600E mutation in CRC essentially excludes Lynch syndrome, with the exception of rare cases associated with *PMS2* germline mutation [18, 19].

### MSI-H colorectal cancers in the era of personalized medicine

CRC is the second leading cause of cancer-related death in the developed world, [20]. Although the response rate of metastatic CRC to the combined chemotherapy is around 50 %, progression of the disease is inevitable and less than 10 % of patients survive >2 years [20]. In adjunct to conventional chemotherapy (e.g. 5-FU, capecitabine, oxaliplatin, irinotecan), metastatic CRC is now treated with a number of drugs aimed at target-specific signaling pathways [e.g. anti-EGFR based therapy (panitumumab and cetuximab for *KRAS*/*NRAS* wild type CRC); bevacizumab (for inhibition of angiogenesis)] [20, 21]. There is an urgent need for more specific predictive markers that will tailor the CRC treatment modalities and improve overall survival in patients with locally advanced and/or metastatic disease.

### Predictive biomarkers of conventional chemotherapy

MSI-H status due to loss of MMR gene function is not only a key player in the pathogenesis of CRC, but is also associated with a different response to classic chemotherapeutic treatment modalities [6].

A seminal clinical study by Ribic et al. [15] revealed the benefit of 5-FU-based adjuvant chemotherapy in patients with stage II and stage III MSI-negative CRC (HR = 0.72, *p* = 0.04) but not in those with MSI-H status (HR = 1.07, *p* = 0.80). Preclinical data also confirmed that tumor cells with MSI-H status are resistant to fluoropyrimidines [e.g. 5-fluorouracil (5-FU) and capecitabine], but may be sensitive to irinotecan and mitomycin C [4, 6, 21]. A meta-analysis by Guetz et al. [22] also highlighted MSI-H status as a strong predictive factor for non-response to 5-FU based chemotherapy. Several enzymes including dihydropyrimidine dehydrogenase (DPD), orotate phosphoribosyl transferase (OPRT), thymidine phosphorylase (TP) and thymidylate synthase (TS) have been associated with the metabolism of the 5-FU pathway [21]. TS is a key enzyme involved in the synthesis of 2′-deoxythymidine-5′-monophosphate, which represents an essential precursor for DNA synthesis [23]. Although data from the available literature are not consistent [(different methodologies, cutoff values), reviewed in Koopman et al.], several studies have found significantly higher expression of TS in MSI-H CRCs, including Lynch syndrome cases, and an association with resistance to 5-FU chemotherapy regimen [24–26] (Fig. 1D). In contrast, MSI-H CRCs appear to be more sensitive to irinotecan which functions as an inhibitor of topoisomerase 1 (TOP1) [4]. Colorectal cancer cell lines exhibit sensitivity to irinotecan when harboring increased *TOP1* gene copy number or increased TOP1/CEP20 ratio [27]. Topoisomerase 1 protein overexpression has also been described in MSI-H CRC [28], although Søndenstrup et al. [29] recently reported an absence of *TOP1* gene copy number gain.

Our results, based on the analysis of both sporadic and hereditary MSI-H and MSI-negative CRCs support the reported differences in TS protein [30] (Fig. 1C; Table 1). TS expression was significantly higher in MSI-H tumors, both sporadic (86 %) and hereditary (100 %), compared to an MSI-negative cohort (31 %, *p* < 0.0001) (Table 1; Fig. 1D). Protein expression of TOP1 trends higher in the Lynch cohort, but is indistinguishable between the sporadic MSI-H and MSI-negative cohorts [30, 31] (Table 1; Fig. 1C).

**Table 1:**
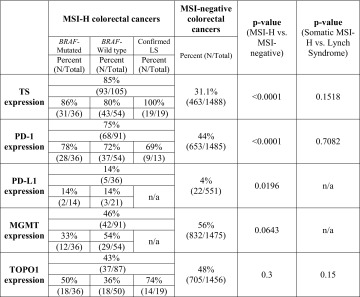
Differential protein expressions in colorectal tumors with different microsatellite instability status

All biomarkers were evaluated using immunohistochemistry

*LS* Lynch syndrome, *MGMT* O6-methylguanine DNA methyltransferase, *MSI* microsatellite instability, *H* high, *Negative* low or stable, *n/a* not available, *PD-1* programmed cell death protein 1, *PD-L1* programmed-death ligand 1, *Topo1* topoisomerase 1, *TS* thymidylate synthase

*p* values were calculated using Fisher-Exact two tail tests

**Fig. 3:**
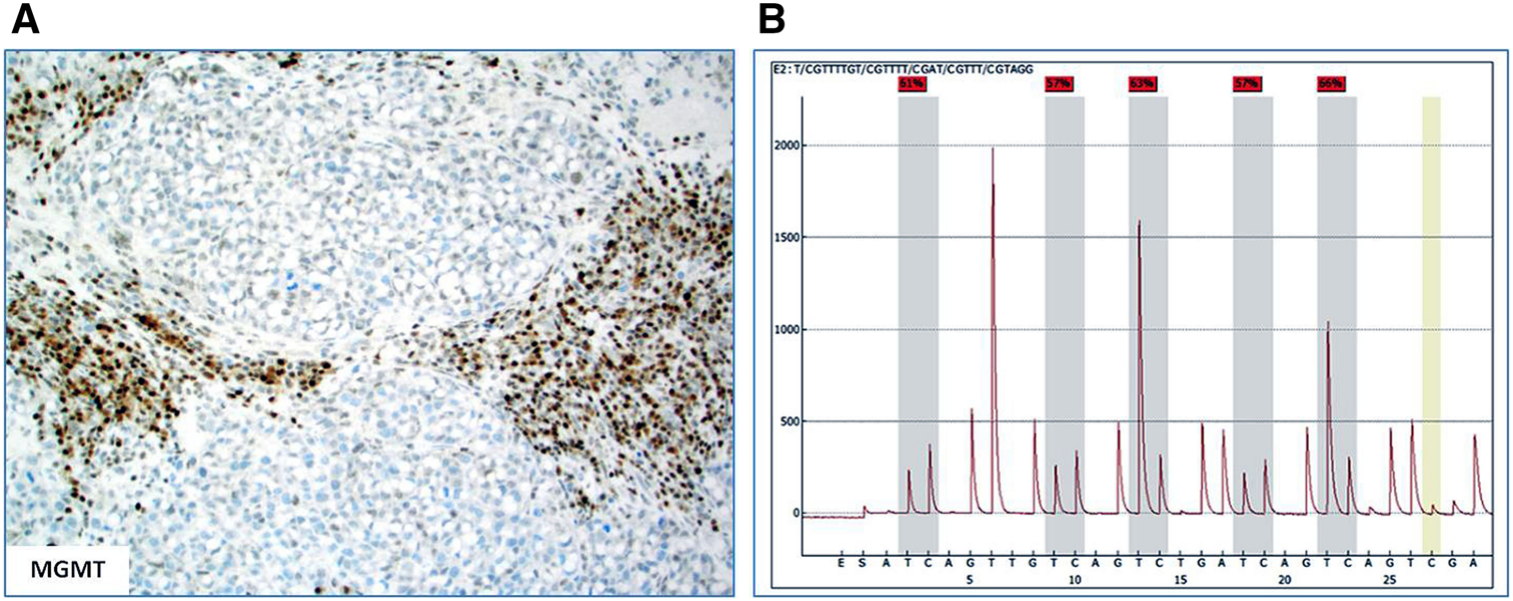
The case from Fig. 2: **A** showing loss of MGMT protein expression by IHC and **B** pyrosequencing results showing hypermethylation of the MGMT promoter (57–66 % methylation at five different sites)

Another biomarker which has been associated with MSI-H CRC is O^6^-methylguanine DNA methyltransferase (MGMT). MGMT is a DNA repair protein with the ability to remove various carcinogenic adducts from the O^6^ position of guanine [32, 33]. Aberrant methylation of the *MGMT* gene promoter occurs in CRCs with the CpG island methylator phenotype (CIMP) [34], and this correlates with loss of *MGMT* expression [32]. In CRC, *MGMT* hypermethylation has been described in subsets of both sporadic and hereditary MSI-H tumors [32, 35, 36] (Fig. 3A, B).

In our cohort of CRC tumors, MGMT protein expression was much lower (33 %) in the sporadic MSI-H cohort compared to MSI-H tumors without *BRAF* mutation (54 %); this difference may be attributable to CIMP in the *BRAF* mutant tumors (Table 1).

MGMT has also been shown to serve as a predictor of response to alkylating agents (temozolomide and dacarbazine) that have been approved for the treatment of various cancers including brain tumors (astrocytoma and glioblastoma multiforme), melanoma, sarcoma, and Hodgkin lymphoma [26, 37]. In colorectal cancer, temozolomide showed limited clinical activity in unselected patient cohorts, but when patients were selected for low expression of MGMT, very promising results were seen [32, 37–40]. However, Karran in his comment [41] pointed out the importance of defects within the mismatch repair machinery in cancer and potential resistance to various chemotherapeutics, including alkylating drugs. In the context of MSI-H CRC and alkylating agents, it is worth noting a study by Hunter et al. [42] who confirmed that inactivating somatic mutations of the *MSH6* gene not only confer a resistance to alkylating agents in brain tumors (gliomas) but also promote tumor growth and progression.

### Next generation sequencing (NGS) profiling in MSI-H

Studies using the currently available NGS platforms allow for investigation into molecular pathways known to contribute to tumorigenesis and progression of CRC and their differential contributions in the setting of sporadic and germline MSI-H CRCs. Several studies reported a frequent disruption of the WNT signaling pathway in Lynch syndrome, with mutations commonly affecting the *APC* and *CTNNB1* (beta-catenin) genes, which are less commonly mutated in sporadic MSI-H CRC [43]. Recent comprehensive molecular profiling of CRC using whole-genome sequencing revealed that the majority (75 %) of hypermutated CRCs exhibited MSI-H status, associated with hypermethylation of *MLH1*, while the remaining 25 % had somatic mismatch-repair gene and polymerase ε (*POLE*) mutations [44]. Along with the expected mutations of *APC, TP53, SMAD4, PIK3CA* and *KRAS*, the study revealed frequent mutations in *ARID1A*, *SOX9* and *FAM123B* genes, while copy-number alterations included amplification of the *ERBB2* and *IGF2* genes. Integrative analysis also indicated an important role for MYC-directed transcriptional activation and repression [44]. Timmermann et al. [45] also reported a significantly higher incidence of mutations in MSI-H than in MSI-negative CRCs.

Le et al. [46] used whole-exome sequencing and showed a mean of 1782 somatic mutations per tumor in patients with mismatch repair-deficient cancer (N = 9) as compared with 73 mutations per tumor in patients with mismatch repair-proficient cancer (N = 6) (*p* = 0.007). Similarly, we performed NGS using a more limited 591-gene panel (available here: http://www.carismolecularintelligence.com/) on 8 MSI-H CRCs, and observed an average of 130.25 mutations per tumor compared to an average of 55.17 mutations per tumor in 189 MSI-negative CRCs (*p* = 0.0004, Student’s *t* test, unpublished data).

### Immune checkpoint proteins PD-1 and PD-L1 in MSI-H colorectal cancer

The PD-1 signaling pathway, composed of the immune cell co-receptor Programmed Death 1 (*PDCD1*, CD279) and its ligands PD-L1 (B7-H1, *CD274*) and PD-L2 (*PDCD1LG2*, B7-DC, CD273), is actively involved in local immunosuppression in human tumors [47]. PD-L1 expression in tumor and associated inflammatory cells has been described in different malignancies, correlating with poor clinical outcome but also with the likelihood of response to targeted immune check point inhibition therapy [48–52]. Several therapeutic monoclonal antibodies inhibiting either PD-1 (nivolumab, pembrolizumab) or PD-L1 (MPDL3280A, Medi4736, BMS-936559) have been developed and are now used for the treatment of various malignancies (e.g. metastatic melanoma, non-small cell lung carcinoma, renal cell carcinoma, bladder carcinoma and Hodgkin lymphoma) [52–54].

In contrast to MSI-negative CRCs, MSI-H CRCs exhibit an active immune microenvironment infiltrated by cytotoxic (CD8+) T-lymphocytes and activated Th1 cells characterized by interferon-γ production and the Th1 transcription factor TBET. This response likely results from the presence of numerous neoantigens (mutated proteins) resulting from the hyper-mutated state of the tumor cells [46, 55–57]. Despite such a “hostile” microenvironment, MSI-H tumor cells are not eliminated by the immune system due to the cancer specific upregulation of various immune inhibitory molecules (checkpoints) including PD-1, PD-L1, Cytotoxic T-lymphocyte-associated protein 4 (CTLA-4), Lymphocyte-activation gene 3 (LAG-3), and Indoleamine (2,3)-dioxygenase (IDO) [31, 55, 58] (Fig. 2E, F; Table 1). These data indicate that MSI-H CRCs are good candidates for checkpoint immunotherapy as recently shown in a small phase 2 clinical trial which included 11 patients with MSI-H CRC. The study showed that the immune-related objective response and immune-related progression-free survival rates were 40 and 78 %, respectively for refractory/and metastatic MSI-H CRC in contrast to MSI-negative CRC (0 and 11 %, respectively) [46]. This led the US Food and Drug Administration to rapidly approve the anti-PD-1 drug pembrolizumab for the treatment of metastatic/refractory MSI-H CRC. Additional studies and clinical trials involving more samples/patients should define the optimal predictive biomarkers for immune checkpoint inhibitors as well as their therapeutic benefits for patients with MSI-H CRC [53].

## Conclusions

Although MSI-H colorectal cancers are heterogeneous (i.e. they can be caused by germline or somatic mutations in different genes), they have similar characteristics that allow them to be grouped together for treatment and clinical management. MSI-H CRCs respond poorly to 5-FU-based chemotherapy (based on thymidylate synthase overexpression), but they may be efficiently treated with camptothecin derivatives (based on topoisomerase 1 overexpression). Hypermethylation/loss of expression of MGMT, which differs between MSI-H cancers based on underlying pathogenic mechanism (i.e. sporadic CIMP MSI-H CRC will be expected to have significantly different MGMT expression from hereditary Lynch MSI-H CRC), may identify a patient subset for clinical investigation. These findings point to the need for individualized profiling of biomarkers and tailoring of therapy for CRCs. Due to an active immune microenvironment and high expression of various checkpoint molecules, MSI-H CRCs are good candidates for targeted immunotherapy with immune checkpoint inhibitors (e.g. pembrolizumab). These cancers also exhibit a distinct hyper-mutated profile that may be amenable to additional treatment options such as vaccination and adoptive-cell-transfer therapy.

## References

[1] Siegel RL, Miller KD, Jemal A (2015). Cancer statistics, 2015. CA Cancer J Clin.

[2] Dienstmann R, Salazar R, Tabernero J (2014). The evolution of our molecular understanding of colorectal cancer: what we are doing now, what the future holds, and how tumor profiling is just the beginning. Am Soc Clin Oncol Educ Book.

[3] Guinney J, Dienstmann R, Wang X (2015). The consensus molecular subtypes of colorectal cancer. Nat Med.

[4] Devaud N, Gallinger S (2013). Chemotherapy of MMR-deficient colorectal cancer. Fam Cancer.

[5] Cheng L (2009) Molecular genetic pathology, 1st edn. Humana Press, New Jersey, pp 455–457.

[6] Hewish M, Lord CJ, Martin SA, Cunningham D, Ashworth A (2010). Mismatch repair deficient colorectal cancer in the era of personalized treatment. Nat Rev Clin Oncol.

[7] Ligtenberg MJ, Kuiper RP, Chan TL (2009). Heritable somatic methylation and inactivation of MSH2 in families with Lynch syndrome due to deletion of the 3′ exons of TACSTD1. Nat Genet.

[8] Valeri N, Gasparini P, Fabbri M (2010). Modulation of mismatch repair and genomic stability by miR-155. Proc Natl Acad Sci USA.

[9] Volinia S, Calin GA, Liu CG (2006). A microRNA expression signature of human solid tumors defines cancer gene targets. Proc Natl Acad Sci USA.

[10] Li GM (2013). Decoding the histone code: role of H3K36me3 in mismatch repair and implications for cancer susceptibility and therapy. Cancer Res.

[11] Li F, Mao G, Tong D (2013). The histone mark H3K36me3 regulates human DNA mismatch repair through its interaction with MutSα. Cell.

[12] Smyrk TC, Watson P, Kaul K, Lynch HT (2001). Tumor-infiltrating lymphocytes are a marker for microsatellite instability in colorectal carcinoma. Cancer.

[13] Gatalica Z, Torlakovic E (2008). Pathology of the hereditary colorectal carcinoma. Fam Cancer.

[14] Maccaroni E, Bracci R, Giampieri R (2015). Prognostic impact of mismatch repair genes germline defects in colorectal cancer patients: are all mutations equal?. Oncotarget.

[15] Ribic CM, Sargent DJ, Moore MJ (2003). Tumor microsatellite-instability status as a predictor of benefit from fluorouracil-based adjuvant chemotherapy for colon cancer. N Engl J Med.

[16] Weisenberger DJ, Siegmund KD, Campan M (2006). CpG island methylator phenotype underlies sporadic microsatellite instability and is tightly associated with BRAF mutation in colorectal cancer. Nat Genet.

[17] Parsons MT, Buchanan DD, Thompson B, Young JP, Spurdle AB (2012). Correlation of tumour BRAF mutations and MLH1 methylation with germline mismatch repair (MMR) gene mutation status: a literature review assessing utility of tumour features for MMR variant classification. J Med Genet.

[18] Funkhouser WK, Lubin IM, Monzon FA (2012). Relevance, pathogenesis, and testing algorithm for mismatch repair-defective colorectal carcinomas: a report of the association for molecular pathology. J Mol Diagn JMD.

[19] Senter L, Clendenning M, Sotamaa K (2008). The clinical phenotype of Lynch syndrome due to germ-line PMS2 mutations. Gastroenterology.

[20] Maurel J, Postigo A (2015). Prognostic and predictive biomarkers in colorectal cancer. From the preclinical setting to clinical practice. Curr Cancer Drug Targets.

[21] Koopman M, Venderbosch S, van Tinteren H (2009). Predictive and prognostic markers for the outcome of chemotherapy in advanced colorectal cancer, a retrospective analysis of the phase III randomised CAIRO study. Eur J Cancer.

[22] Des Guetz G, Schischmanoff O, Nicolas P, Perret GY, Morere JF, Uzzan B (2009). Does microsatellite instability predict the efficacy of adjuvant chemotherapy in colorectal cancer? A systematic review with meta-analysis. Eur J Cancer.

[23] Rose MG, Farrell MP, Schmitz JC (2002). Thymidylate synthase: a critical target for cancer chemotherapy. Clin Colorectal Cancer.

[24] Jensen SA, Vainer B, Kruhoffer M, Sorensen JB (2009). Microsatellite instability in colorectal cancer and association with thymidylate synthase and dihydropyrimidine dehydrogenase expression. BMC Cancer.

[25] Bendardaf R, Lamlum H, Ristamaki R, Korkeila E, Syrjanen K, Pyrhonen S (2008). Thymidylate synthase and microsatellite instability in colorectal cancer: implications for disease free survival, treatment response and survival with metastases. Acta Oncol.

[26] Coppede F, Lopomo A, Spisni R, Migliore L (2014). Genetic and epigenetic biomarkers for diagnosis, prognosis and treatment of colorectal cancer. World J Gastroenterol.

[27] Romer MU, Jensen NF, Nielsen SL (2012). TOP1 gene copy numbers in colorectal cancer samples and cell lines and their association to in vitro drug sensitivity. Scand J Gastroenterol.

[28] Negri FV, Azzoni C, Bottarelli L (2013). Thymidylate synthase, topoisomerase-1 and microsatellite instability: relationship with outcome in mucinous colorectal cancer treated with fluorouracil. Anticancer Res.

[29] Sonderstrup IM, Nygard SB, Poulsen TS (2015). Topoisomerase-1 and -2A gene copy numbers are elevated in mismatch repair-proficient colorectal cancers. Mol Oncol.

[30] Gatalica Z (2014). Thymidylate synthase over-expression underlies the observed lack of 5-FU therapy benefit for MSI-H colorectal cancers. Ann Oncol.

[31] Gatalica Z, Vijayvergia N, Vranic S, Xiu J, Reddy S, Lynch HT, El-Deiry WS (2015) Therapeutic biomarker differences between MSI-H and MSS colorectal cancers. J Clin Oncol 33(Suppl; abstr 3597)

[32] Zheng CG, Jin C, Ye LC, Chen NZ, Chen ZJ (2015). Clinicopathological significance and potential drug target of O6-methylguanine-DNA methyltransferase in colorectal cancer: a meta-analysis. Tumour Biol.

[33] Kaina B, Christmann M, Naumann S, Roos WP (2007). MGMT: key node in the battle against genotoxicity, carcinogenicity and apoptosis induced by alkylating agents. DNA Repair.

[34] Nazemalhosseini Mojarad E, Kuppen PJ, Aghdaei HA, Zali MR (2013). The CpG island methylator phenotype (CIMP) in colorectal cancer. Gastroenterol Hepatol Bed Bench.

[35] Gonzalo V, Lozano JJ, Munoz J (2010). Aberrant gene promoter methylation associated with sporadic multiple colorectal cancer. PLoS One.

[36] Kaz A, Kim YH, Dzieciatkowski S (2007). Evidence for the role of aberrant DNA methylation in the pathogenesis of Lynch syndrome adenomas. Int J Cancer.

[37] Inno A, Fanetti G, Di Bartolomeo M (2014). Role of MGMT as biomarker in colorectal cancer. World J Clin Cases.

[38] Shacham-Shmueli E, Beny A, Geva R, Blachar A, Figer A, Aderka D (2011). Response to temozolomide in patients with metastatic colorectal cancer with loss of MGMT expression: a new approach in the era of personalized medicine?. J Clin Oncol.

[39] Pietrantonio F, de Braud F, Milione M (2015). Dose-dense temozolomide in patients with MGMT-silenced chemorefractory colorectal cancer.

[40] Li Y, Lyu Z, Zhao L (2015). Prognostic value of MGMT methylation in colorectal cancer: a meta-analysis and literature review. Tumour Biol.

[41] Karran P (2001). Mechanisms of tolerance to DNA damaging therapeutic drugs. Carcinogenesis.

[42] Hunter C, Smith R, Cahill DP (2006). A hypermutation phenotype and somatic MSH6 mutations in recurrent human malignant gliomas after alkylator chemotherapy. Cancer Res.

[43] Dionigi G, Bianchi V, Villa F (2007). Differences between familial and sporadic forms of colorectal cancer with DNA microsatellite instability. Surg Oncol.

[44] TCGA (2012). Comprehensive molecular characterization of human colon and rectal cancer. Nature.

[45] Timmermann B, Kerick M, Roehr C (2010). Somatic mutation profiles of MSI and MSS colorectal cancer identified by whole exome next generation sequencing and bioinformatics analysis. PLoS One.

[46] Le DT, Uram JN, Wang H (2015). PD-1 blockade in tumors with mismatch-repair deficiency. N Engl J Med.

[47] Lipson EJ, Forde PM, Hammers HJ, Emens LA, Taube JM, Topalian SL (2015). Antagonists of PD-1 and PD-L1 in cancer treatment. Semin Oncol.

[48] Herbst RS, Soria JC, Kowanetz M (2014). Predictive correlates of response to the anti-PD-L1 antibody MPDL3280A in cancer patients. Nature.

[49] Taube JM, Klein A, Brahmer JR (2014). Association of PD-1, PD-1 ligands, and other features of the tumor immune microenvironment with response to anti-PD-1 therapy. Clin Cancer Res.

[50] Taube JM, Young GD, McMiller TL (2015). Differential expression of immune-regulatory genes associated with PD-L1 display in melanoma: implications for PD-1 pathway blockade. Clin Cancer Res.

[51] Taube JM (2014). Unleashing the immune system: PD-1 and PD-Ls in the pre-treatment tumor microenvironment and correlation with response to PD-1/PD-L1 blockade. Oncoimmunology.

[52] Tumeh PC, Harview CL, Yearley JH (2014). PD-1 blockade induces responses by inhibiting adaptive immune resistance. Nature.

[53] de Guillebon E, Roussille P, Frouin E, Tougeron D (2015). Anti program death-1/anti program death-ligand 1 in digestive cancers. World J Gastrointest Oncol.

[54] Sunshine J, Taube JM (2015). PD-1/PD-L1 inhibitors. Curr Opin Pharmacol.

[55] Llosa NJ, Cruise M, Tam A (2015). The vigorous immune microenvironment of microsatellite instable colon cancer is balanced by multiple counter-inhibitory checkpoints. Cancer Discov.

[56] Topalian SL, Hodi FS, Brahmer JR (2012). Safety, activity, and immune correlates of anti-PD-1 antibody in cancer. N Engl J Med.

[57] Yamamoto H, Imai K (2015). Microsatellite instability: an update. Arch Toxicol.

[58] Gatalica Z, Snyder C, Maney T (2014). Programmed cell death 1 (PD-1) and its ligand (PD-L1) in common cancers and their correlation with molecular cancer type. Cancer Epidemiol Biomark Prev.

